# Mixed-methods study assessing the acceptability and feasibility of Human Challenge Studies for Disease X in Healthy UK Adults in a SARS-CoV-2 pandemic setting

**DOI:** 10.1136/bmjph-2025-003096

**Published:** 2025-12-03

**Authors:** Katherine RW Emary, Rebecca te Water Naude, Arabella Stewart, Maia Patrick-Smith, John A Henry, Marcus English, Tonia M Thomas, Naomi Douglas, Maria Moore, Andrew J Pollard, Samantha Vanderslott, Susanne H Hodgson

**Affiliations:** 1 Department of Paediatrics, University of Oxford, Oxford, Oxfordshire, UK; 2 NIHR Oxford Biomedical Research Centre, Oxford, UK; 3 Jenner Institute, University of Oxford, Oxford, Oxfordshire, UK

**Keywords:** COVID-19, Public Health, Disease Outbreaks

## Abstract

**Introduction:**

Preparation for a future pandemic or serious epidemic caused by a known or yet unknown pathogen (Disease X) is a global health priority. Controlled human infection studies (CHIS) are recognised as a valuable tool to accelerate development of vaccines and immunotherapeutics against pathogens with pandemic potential and may play a role in future pandemic settings. The acceptability of CHIS to potential participants in a pandemic setting has not been explored.

**Methods:**

A mixed-methods study of adults screened to participate in a first-in-human SARS-CoV-2 vaccine trial, using online survey and interviews, was undertaken between September and October 2020 in Oxford, UK. This work assessed individuals’ views, motivations and experiences of participating in a trial during a pandemic setting, including attitudes towards a hypothetical SARS-CoV-2 CHIS.

**Results:**

349 of 770 (45%) invited individuals completed the survey, and 102 survey respondents (29%) participated in a structured interview. Participants were highly educated, research informed and predominantly of white ethnicity. Approximately a third of survey respondents agreed they would be willing to participate in a hypothetical SARS-CoV-2 CHIS. Individuals aged <35 years old, those without children and those who were single were more likely to agree to participate in a hypothetical SARS-CoV-2 CHIS. This decision was dependent on short-term and long-term complications of infection, availability of rescue therapy, likelihood of CHIS to accelerate vaccine development and urgency of the public health need. Motivations to participate in a SARS-CoV-2 CHIS included altruism and a preference to acquire infection of a circulating pandemic pathogen in a controlled setting. The views of family and friends were important in individuals’ decision-making.

**Conclusions:**

Vaccine trial participants are an informed and motivated public group who can provide situated public expertise for CHIS pandemic preparedness. Recruitment to CHIS in a pandemic may be more efficient if targeted to younger, single individuals without children. Media engagement and information specifically for potential CHIS participants’ families is important to facilitate informed discussion.

**WHAT IS ALREADY KNOWN ON THIS TOPIC**

- Controlled human infection studies (CHIS) are valuable tools to accelerate vaccine development.
- Ethical concerns exist regarding the use of CHIS in pandemic settings.
- Study of the acceptability of CHIS in potential participants in a pandemic setting is limited.

**WHAT THIS STUDY ADDS**

- Acceptability of CHIS in a pandemic setting is dependent on short-term and long-term complications for infection, availability of rescue therapy, likelihood of CHIS to accelerate vaccine development and urgency of the public health need.
- Recruitment to CHIS in a pandemic may be more efficient if targeted to younger (<35 years), single individuals without children.
- Family and friends’ opinions are an important influence when individuals are considering volunteering for a CHIS in a pandemic setting. Media engagement and information specifically for close contacts of potential CHIS participants is important to facilitate informed discussion.

**HOW THIS STUDY MIGHT AFFECT RESEARCH, PRACTICE OR POLICY**

- Vaccine trial participants are an informed public group that can provide situated public expertise for CHIS pandemic preparedness.

## Introduction

 Controlled human infection studies (CHIS), where healthy individuals are intentionally exposed to a well-characterised strain of a pathogen at a predefined dose and time, are powerful translational tools, shown to accelerate vaccine development for a number of pathogens.^1 2^ By providing a measure of vaccine efficacy, they can allow iterative revision of vaccine design, prioritisation of candidates for further evaluation, identification of potential correlates of protection and provide insight into mechanisms of pathogenesis and transmission dynamics.^3^ Use of CHIS has grown in recent decades, and such studies are now commonly performed for a variety of bacterial, viral and parasitic infections in populations in low-income, middle-income and high-income settings.^4^ Data from CHIS have supported and accelerated the development of multiple vaccines and contributed key data, leading to the licensure of several vaccines.^5^

Early in the SARS-CoV-2 pandemic, use of CHIS to accelerate vaccine development was proposed and polarised the scientific community and public opinion.^5^,^9^ Traditionally, CHIS were performed using organisms causing mild disease or those fully sensitive to a rescue therapy which could be administered confidently prior to risk of severe disease or long-term sequelae. Indeed, early Academy of Medical Sciences guidelines suggested CHIS was acceptable if the risk of harm was “*minimal*” and “*definitive intervention can take place early in the infection thus making the risk of diseases much lower than that of natural infection*.”^10^

Prior to the SARS-CoV-2 pandemic, there had been no significant consideration of the use of CHIS in a pandemic setting. Early in the SARS-CoV-2 pandemic, the novelty of the organism, lack of understanding of the immediate and long-term risks of infection and absence of an effective treatment meant a SARS-CoV-2 CHIS was considered by some high-risk and ethically unacceptable.^6^ Others, including ‘1DaySooner’, a group originally established to advocate for the conduct of SARS-CoV-2 CHIS,^11^ argued that in the context of a public health emergency, such studies were a moral imperative to accelerate vaccine development and curb mortality rates, and were acceptable, provided participants provided informed consent.^9^

Preparation for a future pandemic or serious epidemic caused by a known or as yet unknown pathogen (Disease X) is a global health priority.^12 13^ Given CHIS are recognised as a valuable tool to accelerate the development of vaccines and immunotherapeutics against pathogens with pandemic potential,^14^ they may play a role in future pandemic settings.^15 16^ The WHO have published guidelines for the ethical conduct of CHIS, including specific criteria for the acceptability of SAR-COV-2 CHIS and the use of CHIS in public health emergency settings.^4 17 18^ These guidelines emphasise the importance of public consultation and engagement when planning CHIS and invited input from organisations and individuals as part of a public consultation. However, of note, no public representatives are credited in the design or review of the guidelines. In a pandemic setting, particularly one involving a novel pathogen with a high mortality rate, public engagement in real time can be contentious as well as practically challenging.^19^,^21^ In addition, identifying and differentiating the views of individuals more likely to participate in a CHIS from the wider public can also be difficult.

In this work, we present data from a mixed-methods study the COVQUAL study, a mixed-methods study of adults who had been screened for eligibility to participate in the UK’s first-in-human (FIH) vaccine trial of the Oxford/AstraZeneca SARS-CoV-2 vaccine. This study assessed individuals’ views, motivations and experiences of participating in a trial during a pandemic setting, including attitudes towards potential SARS-CoV-2 CHIS. Our population and context are novel (box 1); as members of the public who have demonstrated engagement in a FIH clinical trial in a pandemic setting, we suggest they may be a group who are more likely to volunteer for a CHIS and, as such, can provide important insights into the acceptability and feasibility of CHIS in a pandemic setting. This study provides direct evidence of their key concerns, considerations and motivations when contemplating taking part in a hypothetical SARS-CoV-2 CHIS in a pandemic setting. These findings inform current work in pandemic preparedness regarding CHIS and highlight the value of public involvement.

Box 1 **COVQUAL: a mixed-methods study to understand individuals’ motivations for volunteering for a first-in-human COVID-19 vaccine trial and their perception of the risk of participation: A unique study and setting providing insights into a future Disease X pandemic**

**Unique population**

- Healthy UK adults aged 18–55 years who had been screened to participate in phase I SARS-CoV-2 vaccine trial during the SARS-CoV-2 pandemic.
- Self-selecting and supportive of research.
- Accepting of a degree of personal risk—individuals volunteered to participate in a phase I, first-in-human vaccine trial despite being informed of a potential, but low risk of vaccine-enhanced SARS-CoV-2 disease.
- >50% respondents educated to postgraduate level and employed full time.
- 62% of respondents did not have children.
- 20% of respondents had participated in a clinical trial before.

**Unique setting: September 2020–October 2020**

**Figure 3:**
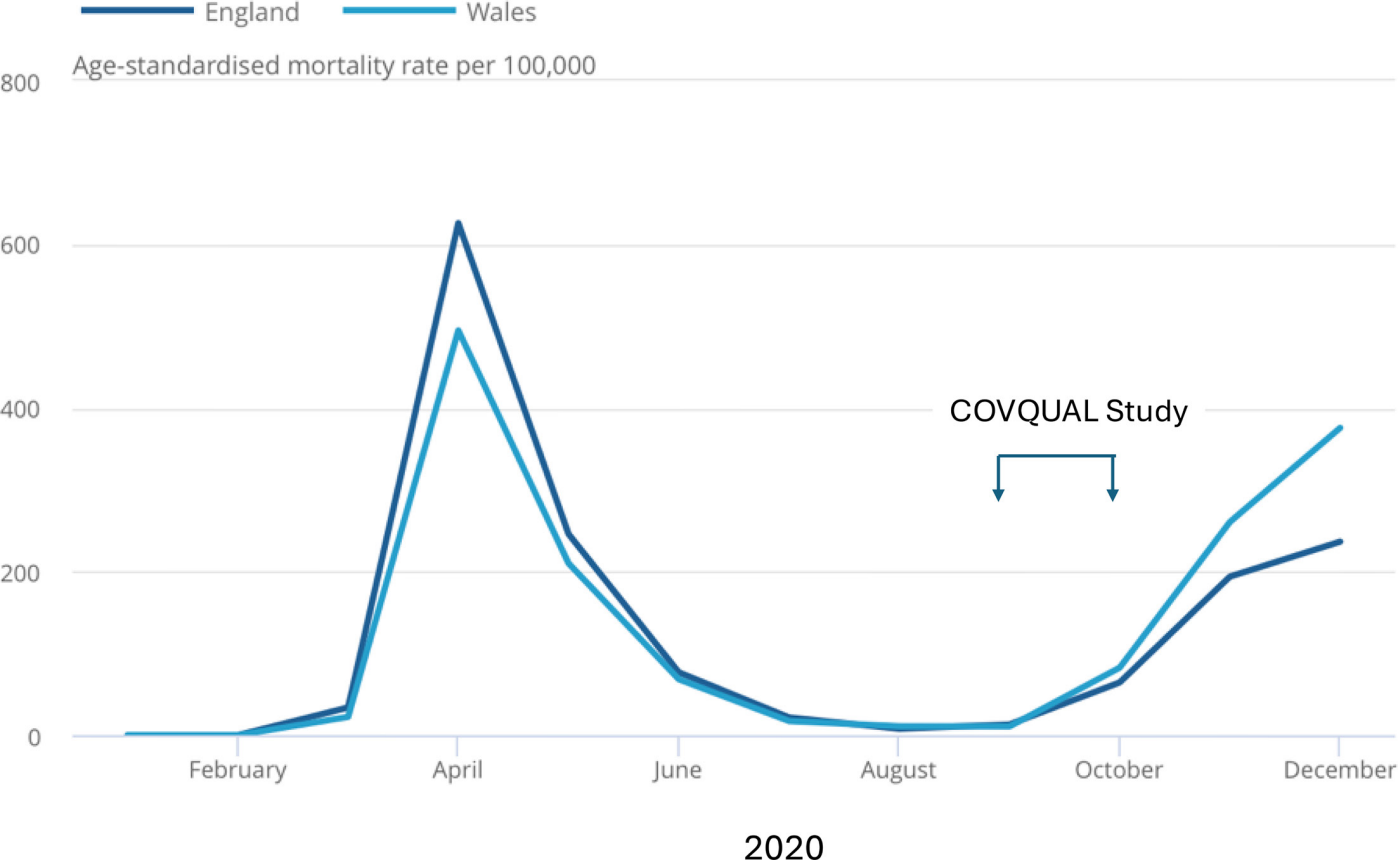
Age-standardised mortality rates (ASMRs) for deaths due to COVID-19 per 100 000 people, England and Wales 2020. Adapted from Office of National Statistics, deaths registered.^49^ Dates of the COVQUAL study (a mixed-methods study to understand individuals’ motivations for volunteering for a first-in-human SARS-Cov-2 vaccine trial and their perception of the risk of participation) are added. During this period, COVID-19 ASMRs were 13.2 and 64.6 per 100 000 in England and 10.8 and 82.7 per 100 000 in Wales in September and October 2020, respectively.

- The UK had experienced one national lockdown (March–June 2020) and restrictions were in place, limiting numbers in social gatherings and mandatory mask wearing. UK SARS-CoV-2 cases had fallen after peaking in the first wave but were beginning to rise again ahead of the UK second SARS-CoV-2 wave (figure 3).
- It was unclear if community transmission rates of SARS-CoV-2 in the UK or elsewhere would be high enough to permit a powered vaccine efficacy study.
- Knowledge of SARS-CoV-2, including risk factors for severe disease (age, ethnicity, occupation) and possibility of ‘long covid’ post-infection, was evolving and discussed in the mainstream media, although the exact nature and risk of this condition were unclear.
- International, high-profile, accelerated research was ongoing to develop vaccines and treatments for SARS-CoV-2 and no SARS-CoV-2 vaccine had been licensed.
  - ChAdOx1 nCoV-19 and other SARS-CoV-2 vaccines had been shown to be safe and immunogenic; however, it was not known whether these vaccine candidates were efficacious in protecting against SARS-CoV-2 disease.
  - SARS-CoV-2 vaccine supply was anticipated to limit roll-out.
  - Dexamethasone and remdesivir were in use as treatments for SARS-CoV-2.
- 1DaySooner, a non-profit organisation to advocate for SARS-CoV-2 controlled human infection studies (CHIS), was established in March 2020, and there was ongoing debate regarding ethics and acceptability of CHIS for SAR-CoV-2, including in the mainstream media.

## Methods

### COV001

COV001 (NCT04324606) is a UK multicentre, FIH, single-blinded, randomised phase I/II study of COVID-19 vaccine ChAdOx1 nCoV-19. This study included healthy volunteers aged 18–55 years. Recruitment started in March 2020, and the first volunteers were enrolled in April 2020. Recruitment and eligibility criteria for the study have been previously described.^22^ Participants enrolled in the study agreed to receive either ChAdOx1 nCov-19 or MenACWY vaccination and attend clinic visits to allow the assessment of vaccine safety and immunogenicity.

### COVQUAL

Individuals who had attended a screening visit to assess their eligibility for participation in COV001 at the Oxford, UK, site and had consented to be contacted about future vaccine-related research (n=771) were invited to participate in a large, mixed-methods study (COVQUAL) to understand individuals’ motivations for volunteering for COV001 and their perception of the risk of participation. Invited individuals included participants who had been enrolled in the trial (ie, those vaccinated) and those who were excluded following screening. The COVQUAL study was independent of COV001. All participants in COVQUAL provided written informed consent for participation in both the survey and interview. COVQUAL research questions were wide ranging about participation in the COV001 study but concentrated on motivations to take part, attitudes to risk and safety, media representation, attitudes to vaccines and experiences as a COV001 trial participant during a pandemic. The COVQUAL survey was designed by the study team with feedback from the Oxford Vaccine Group (OVG) patient and public involvement (PPI) group.

### COVQUAL survey

Individuals were invited via email to complete the COVQUAL survey online, indicating their agreement to survey statements using a Likert scale (online supplemental file 1). Respondents were asked to indicate their agreement to the following statements related to CHIS: “*I would have participated in the trial (COV001) if it also involved; Being infected with COVID-19 and having to isolate from others (Q1), Being exposed to someone who was infected with COVID-19 (Q2) or Being infected with COVID-19 but only with significant financial compensation (Q3*).” Responses to survey statements were coded numerically for data processing: strongly disagree = −2; disagree = −1; neither agree nor disagree = 0; agree = 1; strongly agree = 2. Analysis of the relationship between individuals’ characteristics and survey responses was performed using Kruskal-Wallis test, Dunn’s multiple-comparisons test and Mann-Whitney test as appropriate using Prism V.10.6.0.

### COVQUAL interviews

Survey respondents were invited at the end of the survey to take part in an online semi-structured interview. Online interviews were conducted by a team of 11 researchers (one social scientist (SV), one public engagement manager (TT), three physicians (KRWE, SHH, AS), two research nurses (MM, ND) and four medical students (RtWN, MP-S, JAH, ME).

All researchers conducting interviews received training and used a shared interview guide which included the question: “*Would you agree to be intentionally infected after receiving a COVID-19 vaccine to test if it works (this is called a human challenge or controlled human infection model study)?*” (online supplemental file 2). Interviewers were free to change the wording of this statement, provided the sentiment remained the same. Interviewees were aware that interviewers were researchers working on the COV001 trial at the University of Oxford but did not know any other characteristics or details about the interviewers. Interviews lasted between 20 and 60 min, and no other persons than the researcher and interviewer were present. Field notes were not used, and no repeat interviews were undertaken. Verbal confirmation of consent for audio recordings of interviews using handheld voice recorders was obtained. Audio recordings of interviews were transcribed using an independent transcription company (WayWithWords, https://waywithwords.net/) and analysed by the COVQUAL team using NVivo 12. Interview transcriptions were not returned to participants for review, and participants did not provide feedback on the study findings. A target sample size of 100 participants for interview was set based on both pragmatic and methodological considerations. Qualitative research commonly achieves data saturation with 12–30 interviews.^23^ Given the novelty of the setting and the diversity of the COV001 participant pool, we anticipated a wider range of perspectives. A larger sample size which was feasible within available researcher capacity and timelines was therefore chosen. Participants for interview were recruited sequentially using convenience sampling, according to participant and researcher availability.

A codebook for analysis of interviews was developed iteratively by the trial team. Transcripts were reviewed and coded on an ongoing basis as interviews progressed, allowing the research team to monitor the emergence of new concepts and themes and data saturation (the point at which additional three interviews yielded no further insights or variation within existing themes). Regular meetings were used to check for consistency in the use of codes and gain agreement for new or changed codes. Data collection continued to the planned target to ensure diverse perspectives were captured across participant subgroups.

An inter-coder reliability test showed agreement between three coders (who also performed interviews) was excellent on average, with a kappa value of 0.75+. During coding, any text related to CHIS was coded to a broad ‘challenge’ node. An experiential thematic analysis approach was used by one coder to further analyse the data within this node. NVivo was used to code the text to initial sub-nodes, and these were refined iteratively. The data were extracted using NVivo’s framework analysis function, and the nodes were grouped into themes and further refined.

### Patient and public involvement

OVG’s PPI group provided feedback on the COVQUAL study protocol and participant-facing materials, resulting in modification of the phrasing of survey questions but no other change to the study design.

## Results

The COVQUAL study took place between September and October 2020 in a unique setting (box 1). Data had been published showing the ChAdOx1 nCoV-19 vaccine to be safe and immunogenic;^2^ however, it was not known whether this vaccine or any other SARS-CoV-2 vaccine candidates were efficacious in protecting against disease. The first press release announcing the efficacy of any SARS-CoV-2 vaccine was Pfizer-BioNTech^24^ on 9 November 2020, after all COVQUAL interviews had been completed.

**Figure 1:**
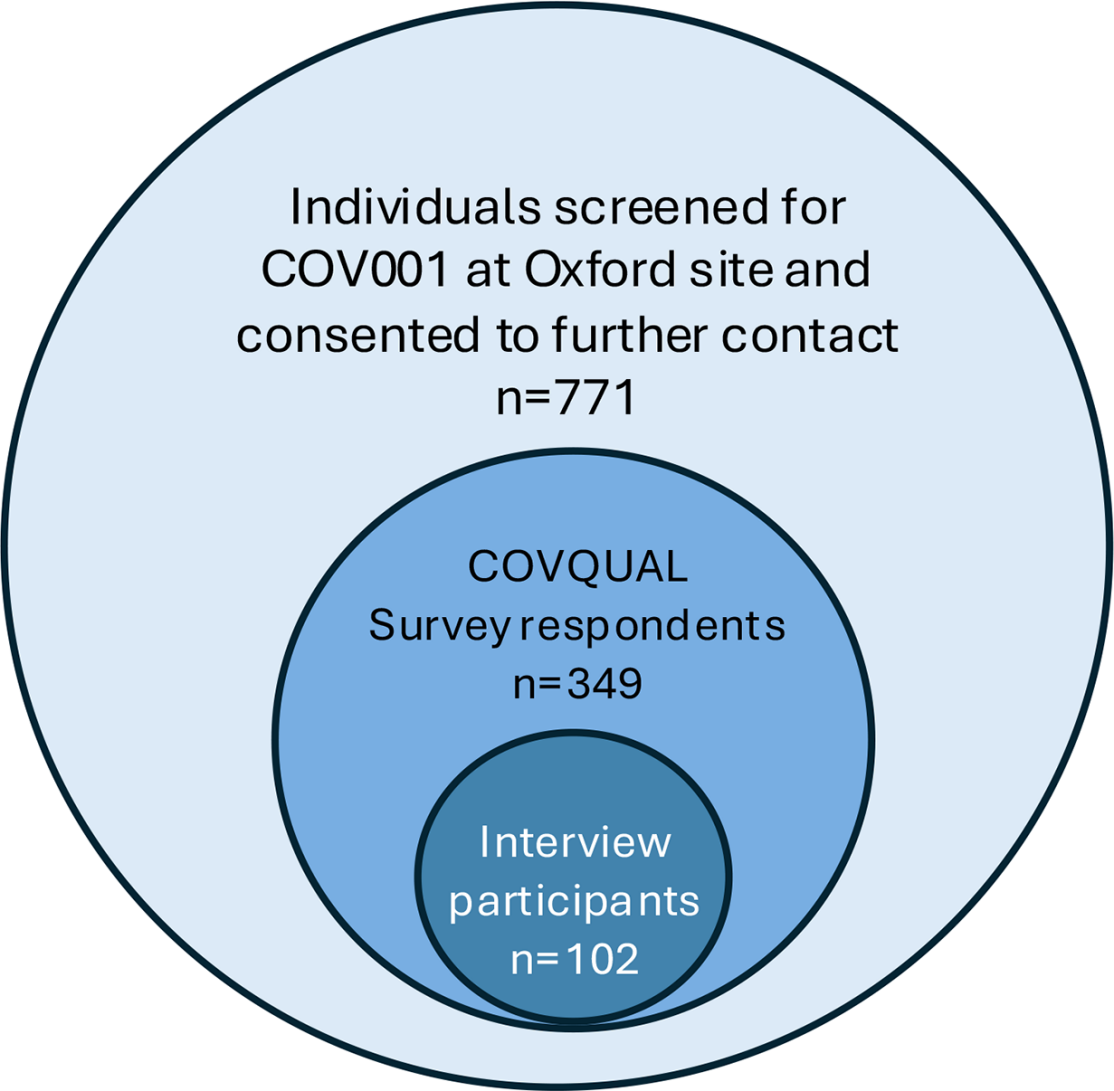
Relationship between COV001 and COVQUAL study participants. COV001 is a UK multicentre, first-in-human, single-blinded, randomised phase I/II study of SARS-Cov-2 vaccine ChAdOx1 nCoV-19. COVQUAL is a mixed-methods study to understand individuals’ motivations for volunteering for COV001 and their perception of the risk of participation.

771 participants who had undergone eligibility screening for participation in the COV001 trial were invited to complete the COVQUAL survey online. Of these, 349 participants completed the survey, 84% (239/349) of which were enrolled and vaccinated as part of COV001 (figure 1). There was no substantial evidence of straight- lining patterns in survey responses. 102 of the survey participants took part in an online semi-structured interview. Characteristics of survey respondents and interviewees have been previously described^25 26^ and were evenly split by sex (female 55%, 191/349), with individuals aged 45–55 years comprising the largest age group (33%, 114/349). A detailed analysis of survey responses and thematic analysis of interview data has been previously published.^25^,^28^ Analysis in this paper focuses on participants’ attitudes to a hypothetical SARS-CoV-2 CHIS in a pandemic setting.

**Figure 2:**
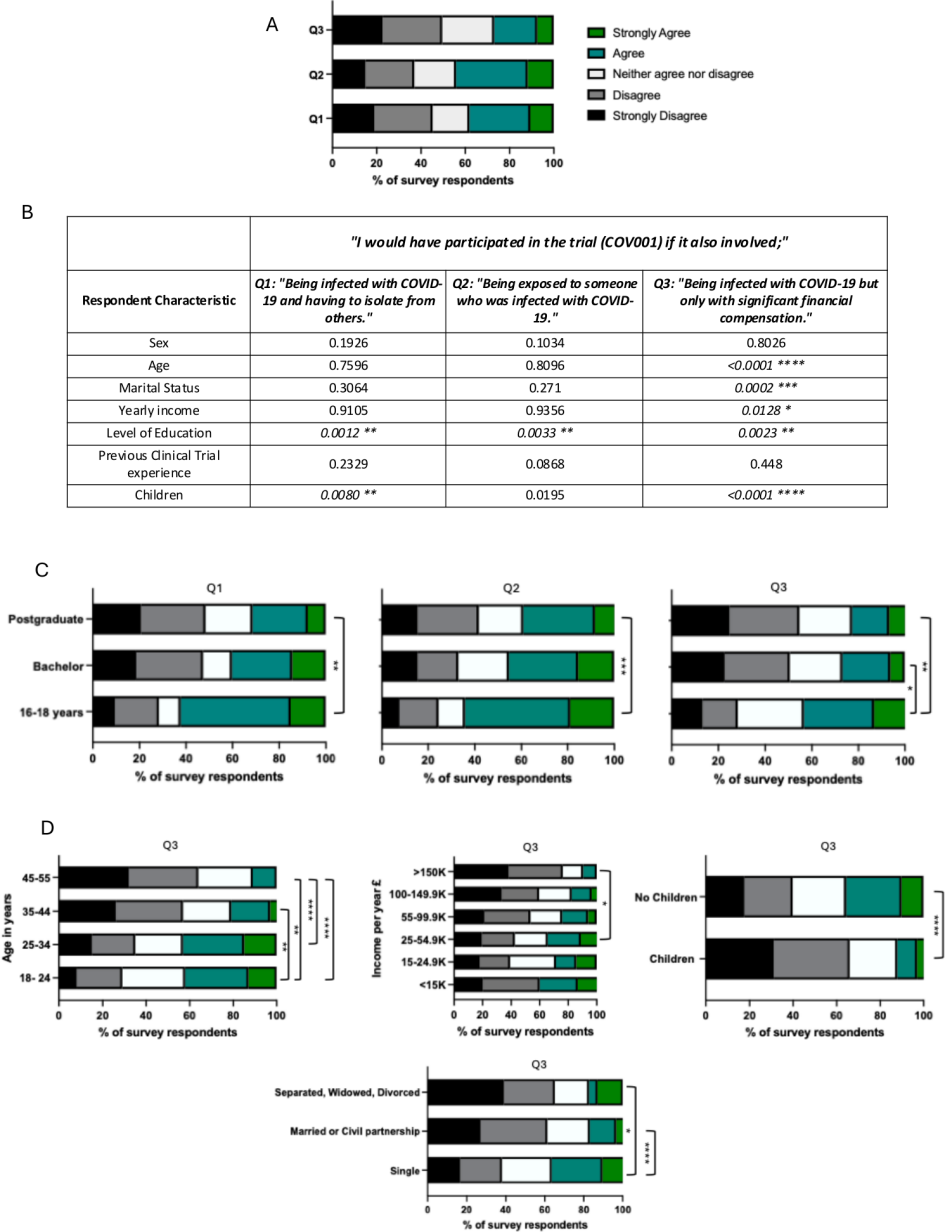
COVQUAL Survey responses. (A) All individuals’ responses to *“I would have participated in the trial (COV001) if it also involved;* Q1: *Being infected with COVID-19 and having to isolate from others*; Q2: *Being exposed to someone who was infected with COVID-19* and Q3: *Being infected with COVID-19 but only with significant financial compensation*.” (B) Analysis of survey responses to Q1, Q2 and Q3 according to participant characteristics using Kruskal-Wallis test for multiple categories (age, martial status, yearly income, highest level of education) or Mann-Whitney U test (sex, children, previous clinical trials experience). (C, D) % survey responses to Q3 presented according to respondents’ age, yearly income, marital status and whether they had children. Statistically significant comparisons using Dunn’s multiple-comparisons test are marked. Participants had the option to select ‘Prefer not to say’ in the survey to questions regarding sex, highest level of education, yearly income, marital status, having children and previous clinical trial experience and these responses were excluded from analysis. *p≤0.05, **p≤0.01, ***p≤0.001, ****p<0.0001*.*

*38*% (133/349) of survey respondents agreed or strongly agreed: “*I would have participated in the trial (COV001) if it also involved being infected with COVID-19 and having to isolate from others*” (Q1). 44% (154/349) agreed or strongly agreed: “*I would have participated in the trial (COV001) if it also involved being exposed to someone who was infected with COVID-19*” (Q2). 26% (91/349) agreed or strongly agreed: “*I would have participated in the trial (COV001) if it also involved being infected with COVID-19 but only with significant financial compensation”* (Q3) (figure 2). Analysis of survey responses according to participants’ reported characteristics showed that individuals’ highest level of education correlated with their responses to all three questions, with individuals who stayed in education up to age 16–18 years (n=53) more likely to agree or strongly agree to Q1 and Q2 than those educated to postgraduate level (n=194) (p=0.0016 and p=0.0046, respectively, Dunn’s multiple-comparisons test), and more likely to agree or strongly agree to Q3 than individuals educated to bachelor’s (n=97) or postgraduate level (p=0.0284 and p=0.0020, respectively, Dunn’s multiple-comparisons test). Participants aged<35 years were more likely to agree or strongly agree with Q3 (p<0.0001, Mann-Whitney test) as were those without children (p<0.0001, Mann-Whitney test) and those who were single rather than married (p<0.0001, Mann-Whitney test).

Analysis of qualitative data collected via interview (n=102) and from optional free-text responses (FTRs) in the survey to Qs1-3 submitted by 25% (88/349) of survey respondents identified four key themes which are explored in the following sections (FTRs are labelled as such, all other quotes are from interviews)

### Understanding of the rationale and value of CHIS in a pandemic setting

During interview and in FTRs, a number of participants demonstrated a clear understanding of the role of CHIS in accelerating vaccine development, especially in the setting of low-community transmission of a pathogen: *“If there was serious questions about the (vaccine) efficacy because say, infection rates national wise were low, and they just were not getting the data that they needed from letting things play out naturally. It seems that yes, it’s risky, but it seems like if you want to test the efficacy of a vaccine, you need to have participants who are in some way exposed to Covid. Whether that’s naturally or through a scientific experiment.*” Interviewees also appreciated the value of the ability to control variables, such as dose and timing of infection in a CHIS, as a means of improving confidence in vaccine efficacy measure: “*Yes, (its) definitely an important thing to do … I guess you need the controlled conditions. So, I think they are a good thing.*” Other interviewees felt that if CHIS could provide a measure of vaccine efficacy more quickly than field efficacy studies, this would be a justification to perform them: *“I would think it would bring about certainty about the vaccine more quickly … And speed is of the essence, isn’t it, at the moment?*”

One interviewee said the exact rationale for CHIS was important and that, while they would be supportive of CHIS to accelerate vaccine development, they would not necessarily support such a study if performed for other reasons they perceived as less justifiable: “*What would be the objective of it? Is it to test the vaccine efficacy, is it for something else? Because I think if it was to give some real positive, clear indication of vaccine efficacy, that is a much bigger incentive than perhaps a more generic study on, I don’t know, understanding something about transmissibility?*” Another participant suggested the graver the consequences of a pandemic, the more acceptable a challenge study was: “*I think, that some of those rules could be changed under careful conditions.*”

Interviewees appreciated the risks were high for a SARS-CoV-2 CHIS and expected trials to be carefully considered and rigorously conducted to high standards: “*It’s not something that I would imagine that anybody would do lightly in terms of you know, a university or something. You wouldn’t do it lightly, and you would have checks and balances, and you would be monitored, all that kind of stuff.*”

### Acceptability of CHIS in a pandemic setting

Some interviewees reported they would not be willing to participate in a SARS-CoV-2 CHIS because of the personal risk of taking part, citing concern about the novelty of SARS-CoV-2 and lack of knowledge about risk factors and the course of infection: “*So you never know how your body’s going to behave with this virus. We’re still not knowing that. So, I think it’s a bit too much risk to expose yourself with a virus which we know not a lot.*” Others highlighted the unpredictable outcome of acute SARS-CoV-2 infection, even in the young and healthy as a concern: “*There’s always these odd cases where you have none of the risk factors, however you still get really ill.*” Numerous interviewees suggested that SARS-CoV-2 CHIS in young, healthy individuals would be more acceptable given the lower morbidity and mortality in this cohort: “*I imagine they’re being very stringent on who they accept in terms of the very, very, very healthy volunteers*.” However, some interviewees appeared to demonstrate an optimism bias, suggesting that their good health and young age might reassure them of an acceptable outcome post-infection: “*My attitude to coronavirus has always been that, actually, I think I would probably be okay. Like I said, I’m slim, I’m fit, I’m healthy, my diet is good, I exercise regularly … I’m 46, but I’m not 56, 66, or 76. I might not be 36, but I’m in a pretty good state, so I think if I got coronavirus, my view has been, I either might be asymptomatic or, at worse, I might feel a bit grotty for a couple of weeks, and that’s it. So, where’s the harm?*”

The unquantifiable risk of ‘long covid’ at the time, absence of long-term follow-up data and the lack of understanding of potential long-term consequences of infection made some feel a CHIS presented an unacceptable personal risk: “*I think the more I find out about COVID and what it can do to people long-term, the less and less keen I think I would be.*”

Some individuals considered the risks of participating in a CHIS higher than participating in an FIH vaccine trial, despite being explicitly warned at the time of screening for the COV001 trial that vaccine-enhanced SARS-CoV-2 disease was a possibility following vaccination (though later found not to be a risk): “(*It’s) one thing to do a vaccine trial where you’re not being deliberately infected, it’s another thing entirely to say, right, we are going to give you this disease that is killing people and don’t think you don’t die*.*”* Several survey respondents commented in FTRs that they would consider taking part in a CHIS but only if they knew they had been randomised to receive the ChAdOx1 nCoV-19 vaccine rather than the control vaccine, MenACWY, suggesting they assumed confidence in the vaccine’s efficacy despite no data yet being available: “*The trial involved placebo, so I wouldn't willingly be exposed to COVID unless I had knowingly received the vaccine … rather than the placebo*.”

Numerous individuals raised the lack of a known cure or guaranteed rescue therapy as a personal reason not to participate, and some even suggested this made SARS-CoV-2 CHIS ethically questionable: “*It doesn’t feel like anyone knows enough about the disease to be able to kind of manage it effectively. I feel like if the sort of treatment was, if there were, well, accepted forms of treatment and you knew that if someone got … COVID-19 and had it really badly but it would be fine because we know that these three steps will cure you of it and you’ll be absolutely fine, then I would support it. But it just feels like you don’t know enough about what, how the virus kind of affects particular people.*” Some FTRs suggested this concern could be mitigated if CHIS participants were guaranteed *“access to medical assistance over and above … local services.*”

Some interviewees suggested that given SARS-CoV-2 was a global public health emergency and individuals were at significant risk of acquiring infection naturally in the community, a SARS-CoV-2 CHIS was more acceptable: *“I mean, we’re all going to be exposed.*” Some suggested that provided risks were mitigated as well as possible and participants provided informed consent, a SARS-CoV-2 CHIS would be acceptable: “*It seems sensible to me that if you could do it as safely as possible, and you’ve got volunteers that understand the risks and are happy with them, why wouldn’t you?*” Indeed, one interviewee suggested acceptability of CHIS to an individual participant was of greater importance than approval of others: *“As long as full consent is given and participants are fully briefed, I don’t really see why anybody should object. If people object, they just don’t have to sign up*.”

### Personal motivations to participate in a pandemic CHIS

Individuals who expressed a willingness to participate in a SARS-CoV-2 CHIS expressed a range of motivations including the wish to be “*doing something useful, doing something proactive instead of waiting around for somebody else to do it for you.*” Some suggested participating in a CHIS would be an exciting and unusual experience they would look forward to sharing this with their loved ones, and this was a motivation to participate: “*I would have been really happy to have done that just because it would have been very exciting. I mean imagine that that would’ve been a great story to tell and I’m excited about experiences and doing unusual stuff. So, I would’ve happily volunteered to do that, that would’ve been great*.”

Other interviewees regarded acquisition of SARS-CoV-2 infection in a CHIS as preferable to that in the community as they felt the closely monitored setting of a clinical study would be safer with lower personal risk than acquiring infection in an unpredictable, ad hoc way in the community: “*At least I’d be in a medical situation.*” Some also saw the opportunity to control the timing and circumstances in which they were personally infected with SARS-CoV-2 as an attractive feature of a CHIS: “*We don’t know whether we’ve been exposed, and you’ve always got that kind of unknown. I prefer a certainty, you can deal with that.*” However, others felt that even in the context of ongoing community transmission, deliberately volunteering to be infected could feel cavalier: “*I think then you do have a slight responsibility perhaps to loved ones and … you are deliberately putting yourself at risk, as opposed to just having a very low-risk vaccine and getting on with your normal life. So, yes, it’s different. I think it is different.*”

Several interviewees saw volunteering for a CHIS as an opportunity to take positive, affirmative action to support vaccine development (“*I just think now because of this whole mess … I just want to do as much as I possibly can if it’s helping towards finding a vaccine*”) and were motivated by the potential to help a large number of people: “*If … results of what I helped with would help … hundreds of thousands of different people, it would be something I would sign up for.*”

### Importance of friends and family

Many interviewees and 23% (20/88) of FTRs referenced the importance of family and friends in influencing their decision to volunteer for a CHIS (“*If it was just for myself, then I’d take the risk, but I think, considering my family’s wishes that I would have to say no*”) and the importance of shared decision-making with family members (“*I’d have to speak to my partner. That would be obviously a joint decision*”). Many individuals highlighted concerns about worrying their family, being away from family members or the potential of spreading the infection to their loved ones: “*In a world where I was single … I would have no qualms with signing up for such a trial …. I live with my partner, and so I would not want to be away from her, I would not want to leave her by herself worrying about me, and I would not want to run the risk of bringing the virus back to her in any way.*” Some individuals suggested that family responsibilities, particularly caring for dependants, would be their deciding factor when considering participation in a SARS-CoV-2 CHIS: “*If it was kind of ten years in the future, or 15 years ago before I had the kids that would have been a different story. But I think there are certain points where you make different decisions, and this is one of them.*” For those with dependants, the possibility of choosing to participate in a study that could possibly result in long-term debilitation, affecting their ability to care for family members was unacceptable: “*I’m a single parent, so I’ve got a 13- and 15-year-old that I’m responsible for. I don’t mind being vaccinated and then by chance being exposed to a virus, but I don’t really want to deliberately incapacitate myself if it can be avoided. Not in my personal circumstances.*” However, several such individuals still recognised the potential role of CHIS to accelerate vaccine development, they just “*want(ed) other people to go first.”*

## Discussion

Global health experts agree the question is not if a future pandemic will occur, but when.^1213 29^,^31^ The precise role of CHIS in pandemic preparedness and pandemic responses is currently unclear but may be significant.^16^ In the case of Disease X, where the pathogen, route of transmission, associated morbidity, mortality and long-term complications are unclear, the role and value of CHIS are understandably difficult to predict. Given that the acceptability and value of CHIS in such settings depend on the potential societal benefits of such studies, risks to individual participants and available alternatives to accelerate vaccine assessment,^4^ the research community will need to be reactive, flexible and considered when contemplating activating a programme of CHIS. Our data support pandemic preparedness surrounding CHIS, informing the ethical and feasibility questions regarding these studies in a pandemic setting.

Since our study, two UK centres have successfully undertaken SARS-CoV-2 CHIS in healthy volunteers.^32 33^ While public engagement work related to this model, including the learning from surveys and focus groups, has been published,^20 21 34 35^ we believe our work to be the first detailed analysis of individuals’ opinions, including one-on-one interviews, on the acceptability of CHIS in a pandemic setting. Taking place early in the pandemic before effective vaccines and rescue therapies had been identified, and including a self-selecting population of individuals motivated to support vaccine research, our study provides detailed insights into a unique population in a unique setting, providing granular detail on individuals’ reasons for participating or not in a hypothetical SARS-CoV-2 CHIS.

Our survey findings show that for a considerable proportion of our study participants (26–44%), taking part in a hypothetical CHIS in a pandemic setting was considered acceptable. This finding is similar to that reported in another mixed-method study performed at the same time using a broader UK population^20^ and supports findings from other survey and focus group-based studies from high-income settings showing support for CHIS in the SARS-CoV-2 pandemic.^21 35 36^

Our participants were able to clearly articulate the rationale for CHIS in such settings and provided insightful comments on what they felt would be acceptable and unacceptable scenarios of such studies, for example, supporting CHIS if community transmission of a pathogen were too low to allow determination of vaccine efficacy from field studies, and questioning their value if they did not lead to a tangible acceleration of deployment of an effective vaccine. However, it is important to note the unusual cohort from which our subjects were drawn. The majority of our participants were highly educated, research literate and had already engaged with a research initiative with some inherent personal risk by volunteering to participate in an FIH of a SARS-CoV-2 vaccine. Of interest, the less higher education individuals had completed, the more likely they were to support participating in a hypothetical CHIS. Our study did not provide participants with a detailed explanation of the risks of taking part in a CHIS, and it may be that participants with greater higher education experience were more aware of such risks and this influenced their survey responses. Familiarity with and confidence in the University of Oxford, which is known to have considerable experience in CHIS, may have influenced participants’ views.^27^ Indeed, this consideration is a key reason why future CHIS in pandemic settings should be led by experienced centres who can canvas existing databases of motivated and informed individuals to identify potential CHIS participants and are experienced in obtaining informed consent for potentially controversial trials.

There was clear alignment between our survey and interview data on several themes, including the finding that younger individuals, under 35 years old, were more likely to be willing to participate in CHIS in a pandemic setting. The reasons for this are likely multifactorial due to the lower mortality of SARS-CoV-2 associated with younger age known at the time, the lower likelihood of younger individuals having dependants and the fact that younger adults may be more likely to choose the risky option for negatively framed high-amount mortality-based decision scenarios than older adults.^37^ Having children was a key factor that influenced participants’ likelihood of volunteering for CHIS, with both survey and interview data showing responsibility to care for dependants and support loved ones as important reasons that could make individuals less likely to volunteer for a CHIS. Such findings can help inform targeted, future recruitment strategies to increase the efficiency of enrolment to future CHIS in a pandemic setting.

Our results emphasise the importance of the opinion of family and friends in determining individuals’ likelihood of participating in a CHIS. This reinforces the important role of the media in communicating information about CHIS to the broader public,^27 38^ and the value of conducting comprehensive community engagement ahead of implementation to ensure messaging is coherent and accessible, not just for potential participants but also their loved ones. Indeed, the creation of materials specifically for close contacts of potential participants in CHIS could be a valuable means of supporting informed discussions between potential participants and their families, which may be particularly challenging in a pandemic setting where there are many unknowns. We include this and other learning points from our findings for the conduct of future CHIS in a pandemic setting in box 2.

Box 2 **Learning points for future controlled human infection studies (CHIS) in a Disease X pandemic setting**

- Vaccine trial participants are a motivated and informed public group that can provide situated public expertise for CHIS pandemic preparedness.
- Depending on the context, CHIS in a pandemic setting may be acceptable for some individuals even in the absence of an established rescue therapy or efficacious vaccine.
- Recruitment to CHIS in a pandemic may be more efficient if targeted to younger, single individuals without children.
- Family and friends’ opinions are an important influence when individuals are considering volunteering for a CHIS in a pandemic setting. Media engagement and information specifically for participants’ families is important to facilitate informed discussion.

To be ethically acceptable, research including human subjects must carefully consider the risks and benefits of participation to individuals. Traditionally, institutional review boards have suggested “*clinical research is justified only when participants are protected from excessive risks*.”^39^ In the context of CHIS, participants rarely directly derive benefit from involvement, instead contributing to societal good by advancing prophylactic or therapeutic interventions.^40^ In the setting of CHIS in a pandemic, the risks to participants are greater, but so are the potential benefits to society.^4^ Indeed, this altruistic motivation was a key factor cited by subjects who were supportive of volunteering in principle for a SARS-CoV-2 CHIS. While some individuals questioned whether the lack of a rescue therapy made a CHIS ethically acceptable, others suggested that provided participants gave informed consent and fully appreciated the risks of taking part, a SARS-CoV-2 CHIS was acceptable. While the upper limit of acceptable risk for research remains debated,^41^,^43^ it is an accepted tenet that participants must be able to provide informed consent to take part in research.^44^ To do this, individuals must fully understand the risks of participation, which in the case of CHIS in a pandemic setting may be unknown. In our setting, participants appeared informed about SARS-CoV-2, and some acknowledged the possibility of unknown complications following infection. However, it remains unclear whether individuals can truly give informed consent if the risks are unknown^45^ and earlier in another pandemic scenario where less is known about the pathogen, it is unclear if individuals would be as willing to participate or could provide informed consent. These are multifaceted ethical issues, but our findings demonstrate that potential CHIS participants can appreciate such nuances and can provide perceptive insights into such debate. Indeed, our work shows that vaccine trial participants are an important resource when considering CHIS, not only for understanding the motivations of potential CHIS participants, but to provide insights into the acceptability of CHIS and opinions of the wider society researchers serve. Our participants voiced many of the issues addressed in recent WHO guidelines for the ethical conduct of CHIS^4^ exemplifying the value of public expertise as a key resource to input into future guidelines and early CHIS planning.^16^

The strengths of our work include the novelty of the setting and population, the relatively large number of individuals who contributed to the qualitative data and the concordance between findings from survey and interview data. However, there are several important methodological limitations that should be considered when interpreting our findings.

1. **Sample characteristics and representativeness:** COVQUAL participants were a convenience sample drawn from individuals screened for the COV001 clinical trial in Oxford, UK. This group was generally highly educated, predominantly white, employed and familiar with research. Consequently, our findings may not be representative of the broader UK population or of more ethnically and socioeconomically diverse groups, limiting generalisability.
2. **Context and timing of data collection:** At the time of the COVQUAL study, a SARS-CoV-2 CHIS was being contemplated by researchers but had not yet been implemented, so our participants’ responses were necessarily based on a hypothetical scenario. We cannot know if their responses would hold true for an actual CHIS, nor how these views may have evolved as knowledge and experience increased during the SARS-CoV-2 pandemic.
3. **Sampling and qualitative depth:** Our survey sample was large (n=349), but only a subset participated in qualitative interviews (n=102). While this exceeds typical qualitative sample sizes for achieving data saturation,^46 47^ our data represent only a fraction of the potential participant pool, which may have constrained the diversity of perspectives captured.
4. **Researcher involvement and subjectivity:** Multiple interviewers and coders were involved in data collection and analysis. Despite structured interview guides, regular team discussions and cross-checking of coding, minor variations in phrasing and interpretation between researchers could have influenced participants’ responses and thematic analysis.
5. **Mixed-methods design considerations:** Triangulation of survey and interview data strengthened the depth and credibility of our findings but introduced limitations.^48^ The two components did not always address identical questions, restricting direct comparability across data sources.
6. **Survey instrument clarity:** Given the rapid development of the COVQUAL study during the SARS-CoV-2 pandemic, some survey questions could have been clearer. For example, Q3 combined willingness to participate in a CHIS with financial compensation, making it difficult to disentangle intrinsic acceptability from potential financial motivation.

Despite these limitations, our opportunistic study notably expands the body of research on the feasibility and acceptability of CHIS in a pandemic setting and uniquely provides detailed qualitative data directly from a cohort of individuals who may be more likely to consider participating in a CHIS. Our work demonstrates that it is possible to rapidly collect individuals’ detailed perspectives during a pandemic and that such input can be valuable and nuanced. While our findings are context dependent and specific to the SARS-CoV-2 pandemic, they do provide important agnostic learning points for pandemic preparedness and recruitment to CHIS in a pandemic setting.

## Supplementary material

10.1136/bmjph-2025-003096 online supplemental file 1

10.1136/bmjph-2025-003096 online supplemental file 2

## Data Availability

Data are available upon reasonable request.
